# Minimizing eddy currents induced in the ground plane of a large phased-array ultrasound applicator for echo-planar imaging-based MR thermometry

**DOI:** 10.1186/s40349-016-0047-x

**Published:** 2016-02-03

**Authors:** Silke M. Lechner-Greite, Nicolas Hehn, Beat Werner, Eyal Zadicario, Matthew Tarasek, Desmond Yeo

**Affiliations:** Diagnostics, Imaging and Biomedical Technologies Laboratory, GE Global Research Europe, Garching n., Munich, Germany; IMETUM, Technical University Munich, Garching n., Munich, Germany; Center for MR-Research, University Children’s Hospital Zurich, Zurich, Switzerland; InSightec Ltd., Tirat Carmel, Israel; Diagnostics, Imaging and Biomedical Technologies Laboratory, GE Global Research Niskayuna, Albany, NY USA

**Keywords:** MR thermometry, Focused ultrasound, Eddy currents, Proton resonance frequency shift, Echo-planar imaging, Phased-array transducer

## Abstract

**Background:**

The study aims to investigate different ground plane segmentation designs of an ultrasound transducer to reduce gradient field induced eddy currents and the associated geometric distortion and temperature map errors in echo-planar imaging (EPI)-based MR thermometry in transcranial magnetic resonance (MR)-guided focused ultrasound (tcMRgFUS).

**Methods:**

Six different ground plane segmentations were considered and the efficacy of each in suppressing eddy currents was investigated in silico and in operando*.* For the latter case, the segmented ground planes were implemented in a transducer mockup model for validation. Robust spoiled gradient (SPGR) echo sequences and multi-shot EPI sequences were acquired. For each sequence and pattern, geometric distortions were quantified in the magnitude images and expressed in millimeters. Phase images were used for extracting the temperature maps on the basis of the temperature-dependent proton resonance frequency shift phenomenon. The means, standard deviations, and signal-to-noise ratios (SNRs) were extracted and contrasted with the geometric distortions of all patterns.

**Results:**

The geometric distortion analysis and temperature map evaluations showed that more than one pattern could be considered the best-performing transducer. In the sagittal plane, the star (d) (3.46 ± 2.33 mm) and star-ring patterns (f) (2.72 ± 2.8 mm) showed smaller geometric distortions than the currently available seven-segment sheet (c) (5.54 ± 4.21 mm) and were both comparable to the reference scenario (a) (2.77 ± 2.24 mm). Contrasting these results with the temperature maps revealed that (d) performs as well as (a) in SPGR and EPI.

**Conclusions:**

We demonstrated that segmenting the transducer ground plane into a star pattern reduces eddy currents to a level wherein multi-plane EPI for accurate MR thermometry in tcMRgFUS is feasible.

## Background

Transcranial magnetic resonance (MR)-guided focused ultrasound (tcMRgFUS) has become a promising technology for non-invasive treatment of several types of brain diseases and for functional neurosurgery in particular [1–4]. Unlike established treatment options such as deep brain stimulation, tissue resection, or RF ablation, tcMRgFUS does not require invasive procedures that carry high risks of complications including infection and hemorrhages. Furthermore, in contrast to non-surgical cancer treatment modalities like radiation therapy, tcMRgFUS does not use ionizing radiation. This allows for multiple treatment sessions without increased risks of collateral damage to remaining healthy tissues.

In tcMRgFUS, treatment monitoring is achieved by MR temperature mapping, using techniques that make use of the temperature-dependent proton resonance frequency shift (PRFS) phenomenon [5–9]. Today, only single slice phase images from a gradient echo-based (GRE) sequence are acquired at the location of the hot spot [10] with a temporal resolution of about 3 to 5 s. To increase the safety of tcMRgFUS clinical procedures, the spatial coverage of the temperature maps should be increased and multi-slice thermometry should be employed. This is especially important because of the risk of unintentional heating in areas outside the targeted region, e.g., through acoustic energy absorption in the skull [11, 12] or secondary acoustic foci. Increasing the spatial coverage of MR thermometry, however, may degrade the temporal resolution of temperature monitoring. As such, fast MR thermometry techniques are highly desirable in clinical tcMRgFUS to reduce acquisition time and, thus, increase spatial coverage of the hot spot.

One approach to increase temporal resolution is to use fast imaging sequences such as multi-shot gradient echo EPI for fast multi-plane image tracking and MR thermometry [13–17]. As one of the fastest imaging methods, EPI facilitates very fast 3D hot spot localization when used in MR thermometry. EPI also has other applications related to treatment planning for tcMRgFUS applications. It is the most commonly used MR imaging sequence for brain functional MRI (fMRI) [18] and diffusion-weighted imaging [19], which can provide critical information for pre-surgical treatment planning [20, 21] and post-interventional evaluations [2]. For example, Köhler et al. [13] and Mougenot et al. [14] used multi-shot EPI for fast multi-plane image tracking in MR thermometry where up to six slices could be acquired in the same time frame.

Clinically desirable spatial and temporal resolution can potentially be achieved with EPI. However, EPI is prone to imaging artifacts, such as *B*_0_-inhomogeneity-induced geometric distortions. In addition, the increased activity of switching gradients inherent in this sequence induces undesired eddy currents, which in turn generate secondary magnetic fields that disrupt the carefully constructed arrangement of time-varying magnetic fields for spatial localization of nuclear spins. This induces geometrical distortions in the reconstructed images.

**Fig. 1:**
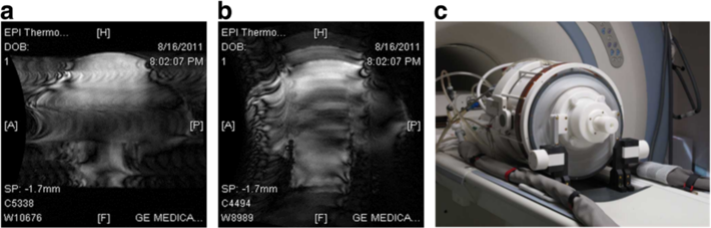
Magnitude image of an EPI scan (**a**) with phase encoding direction in anterior/posterior direction of a sagittal slice of a gel phantom mounted inside the transducer setup (**c**). The distortions also occur when changing the readout direction from a head to foot direction (**b**). **c** Phased-array transducer setup with a dedicated eight-channel phased-array receive coil and water cooling pipes for circulating water through the transducer

In a typical ultrasound applicator, among other conductive structures, a conductive ground plane is often present. During an EPI sequence, gradient-induced eddy currents may occur in this ground plane, which could cause significant geometrical distortions in the EPI images. Such distortions markedly degrade the spatial fidelity and accuracy of MR temperature mapping during tcMRgFUS procedures (Fig. 1a, b). To date, only eddy currents induced during RF excitation are compensated for in the research area of cryo-ablation [22, 23]. Dragonu et al. [24] proposed real-time geometric distortion correction based on field maps for gradient echo-recalled EPI images by considering off-resonance effects. A similar technique was applied by Samoudi et al. [25] where they added gaps to tungsten collimator geometries for single-photon emission computed tomography to reduce eddy currents induced by switching gradients of the MR system.

For the tcMRgFUS setup described here, the gradient-induced eddy currents distort the readout gradients, resulting in image artifacts and geometric distortions of the object under investigation. The eddy currents, which form primarily on the conductive material that serve as the transducer electrode ground plane, give rise to spatio-temporal variations of static magnetic field in the imaging field-of-view (FOV). These variations in the magnetic field induce non-linear geometric distortion in the MR images, which can significantly degrade image quality for temperature mapping.

Here, we investigated the impact of gradient-induced eddy currents on the accuracy of multi-plane EPI-based temperature maps in the presence of the transducer electrode ground plane of a tcMRgFUS applicator by first performing finite element electromagnetic field (FEM) simulations to calculate the eddy current distribution on the ground plane and the reduction potential of certain segmentation patterns. Inspired by these results, different segmentation patterns were experimentally tested by acquiring (i) spoiled gradient echo scans (SPGR) and (ii) multi-shot EPI scans to consider all influencing aspects of a commercial MR scanner on a transducer mockup model. The results will help the transducer designers to improve MR compatibility of future transducers without a necessary high degree of segmentation. Elements of this work were presented at the 2012 [26] and 2015 [27] ISMRM meetings, respectively, where we propose a re-segmentation of the focused ultrasound transducer array ground plane into a star pattern to enable fast multi-plane temperature tracking.

## Methods

Eddy currents are currents in conducting structures induced by fast-switching gradient fields that cause time-dependent field disturbances. The eddy currents create a secondary magnetic field that counteracts the desired field. Typically, it is assumed that eddy current dynamics behave reproducibly in space and time. The gradient and eddy current field can be expressed by a truncated spherical harmonics series referred by spherical harmonic decomposition [28]. In a certain region of interest, it is assumed that the eddy current field changes linearly. This assumption is the basis for eddy current compensation called gradient pre-emphasis as implemented in clinical MR scanners [29]. Other eddy current minimization techniques are targeted during gradient coil design phase [26] or by playing out suitably parameterized compensation pulses. For diffusion-weighted EPI for example, compensation of higher-order eddy current terms is mandatory [30]. The goal of eddy current compensation is to minimize the induction of eddy currents in an MR system such as the cryostat of the magnet. However, in tcMRgFUS, a transducer system is placed inside the FOV of the MR system. Hence, any conducting structure present in the transducer will cause additional eddy currents not anticipated by the calibration of the compensation parameterization. The induced eddy currents have certain strength; they also persist for a duration that depends on how fast the gradient field changes, its field strength, and the properties of the conducting material (e.g., conductivity, thickness, and skin depth) that characterize the depth of magnetic field penetration [29]. Here, we investigate the impact of gradient-induced eddy currents in the transducer electrode ground plane, using FEM simulations and phantom experiments.

MR temperature mapping was performed based on the PRFS method [6, 7]. In general, a proton’s resonance or Larmor frequency *ω*_0_ is defined by the product of the gyromagnetic ratio *γ* and externally applied magnetic field *B*_0_. The precise Larmor frequency of a given proton is influenced by its atomic environment because any magnetic spins (electron or other magnetic nuclear spins) near it will generate small magnetic fields that add or subtract from *B*_0_. The change in the resonance frequency due to a change in temperature can be assessed by measuring the change in accrued phase in a series of GRE images. The temperature change Δ*T* can be extracted by subtracting the phase images *φ*_*T*_ during sonication from a baseline image *φ*_*T*0_ that was acquired before sonication according to [31]

1 $$ \Delta T=\frac{\varphi_T-{\varphi}_{T0}}{\gamma \alpha {B}_0TE}, $$

where *α* is the PRFS temperature coefficient. The PRFS method is commonly used for MR thermometry because it is a simple, robust MR thermometry method in water-based tissues.

### Simulation setup

**Fig. 2:**
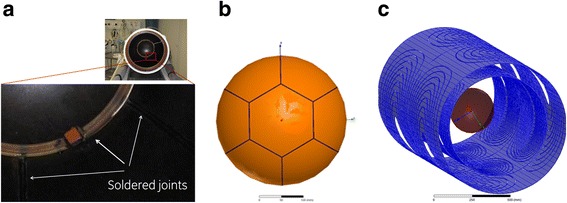
**a** Picture of the ground plane and a close up where the soldered joints are highlighted with the *white arrows*. **b** Picture of a CAD model of the copper layer of the ground plane, illustrating the soldering of seven segments to a continuous surface. The CAD model in **b** is used in FEM simulations to predict the induced eddy currents on its surface. **c** Schematic of the FEM model (full shield and primary *y*-coil) of the depicted MRI system. The hemisphere in the iso-center demonstrates the positioning of the transducer ground plane inside the gradient coil

**Fig. 3:**
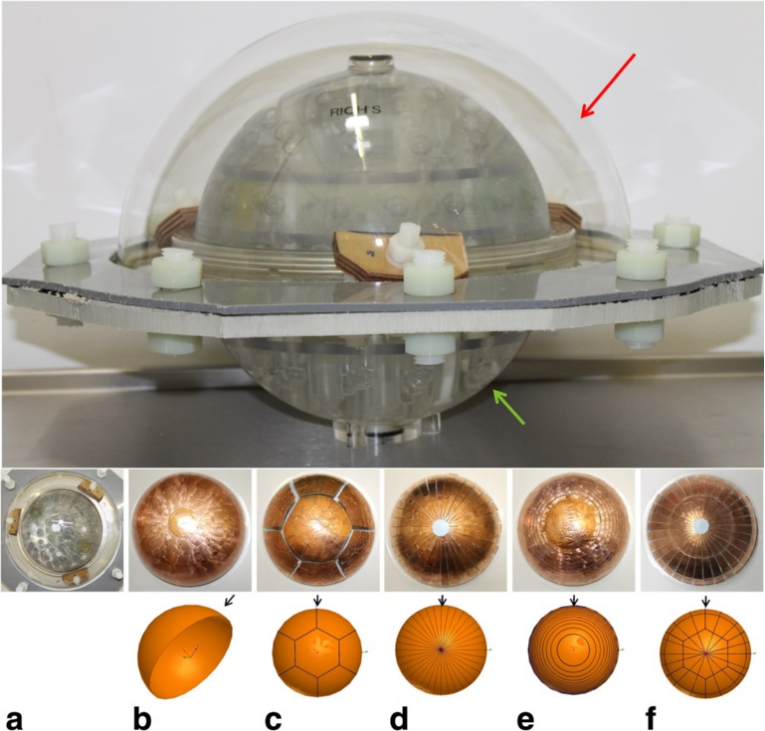
(*Top*) Picture showing transducer mockup model containing reference plastic hemisphere (*red arrow*) and ADNI phantom (*green arrow*). (*Middle*) Pictures of different copper patterns attached to the outside of the hemisphere: **a** reference without copper sheet, **b** solid copper surface (one segment, average surface of segment ≈1413 cm^2^), **c** seven-segment clinical pattern (average surface of segments ≈199 cm^2^), **d** star pattern (32 segments, average surface of segments ≈37 cm^2^), **e** ring pattern (16 segments, average surface of segment ≈92 cm^2^), and **f** a combined star-ring pattern (64 segments, average surface of segment ≈27 cm^2^). (*Bottom*) Modeled patterns of FEM simulations with 36 (**d**), 17 (**e**), and 54 segments, respectively. *Black arrows* indicate the points of view of the ground planes when plotting the current densities in Fig. 4

FEM-based magnetic field simulations using the electromagnetic field simulation software Maxwell3D (Ansys, Canonsburg, USA) were performed to study the eddy currents induced on the surface of the transducer ground plane and to test the role of the copper ground plane in causing the image distortions. Maxwell3D solves for Maxwell equations using the finite element method to solve for static, frequency domain, and time-varying electromagnetic fields. More information on the Maxwell3D software can be found online [32]. The theory is described in detail in [33, 34]. The 3D simulation model included the *y*-gradient coil of an MR scanner only (GE Signa Excite II 3.0 T, General Electric, Milwaukee, USA) with a maximum gradient strength and slew rate of 49.5 mT/m and 150 T/m/s, respectively (Fig. 2c). Following the basic design of the InSightec ultrasound applicator (Imasonic, Imasonic SAS, Voray sur l’Ognon, France), a 0.25-mm thick copper hemisphere, 30 cm in diameter, represented the ground plane and was modeled at the iso-center of the gradient coil. The magnitudes of the eddy currents induced in a conducting structure vary with the surface area of the structure. Interrupting the flow minimizes the currents and hence the magnetic field distortions. This is typically done in RF shield design [35]. In simulation, five different ground plane segmentation patterns were designed to modify the eddy current flow; these patterns were evaluated with respect to active imaging gradients. The designed patterns were a full copper hemisphere, a segmented hemisphere similar to the real setup, a star pattern, a ring pattern, and a star-ring pattern (hereafter labeled as cases (b), (c), (d), (e), and (f), respectively, in the text and figures). According to the manufacturer, a technical realization of segmenting the transducer into patterns (d), (e), and (f) is possible without any technical restrictions. The different segmented ground plane models are shown in the third row of Fig. 3. The electrical conductivity of the patterns was set to that of copper in the simulations (*κ* = 5.8 × 10^7^ S/m).

For reference, a simulation was performed in magneto-static solving mode to replicate the situation without the copper ground plane (hereafter labeled as case (a) in the text and figures). The extracted fields inside a FOV with a diameter of 20 cm represent the gradient field without eddy currents (*B*_ref_). In a second simulation, the gradient coil model is pulsed in frequency solving mode where the induced eddy currents on the surface of the copper ground plane and the resulting counteracting magnetic fields are considered in the field solution. The extracted fields represent the gradient field with eddy currents (*B*_ec_). The frequency solving mode expects at least one frequency for which the model is solved and which goes into the skin depth calculation describing how deep the induced currents penetrate into the conducting structure. Here, a frequency of 700 Hz was chosen. The value was determined by Fourier transforming the time-dependent *x-* and *y*-gradient waveforms of the EPI pulse sequence as prescribed in Table 1 and by calculating the full width at half maximum frequency.

**Table 1:** A typical clinical protocol for SPGR-based temperature mapping and showing SPGR and EPI sequence parameters used for experimental evaluation in this study

	SPGR—clinical protocol	SPGR	EPI
Plane	Sagittal/axial	Sagittal/axial/coronal	Sagittal/axial/coronal
Mode	2D	2D	2D
Protocol	SPGR, seq-fast-MPh	SPGR, seq-fast-MPh	Gradient echo EPI, fast-MPh
FOV	350 mm	350 mm	350 mm
Slice thickness	3.0 mm	4.7 mm	4.7 mm
Frequency direction	S/I (sagittal), R/L (axial)	S/I (coronal, sagittal), R/L (axial)	S/I (coronal, sagittal), R/L (axial)
Frequency resolution	256	144	144
Phase resolution	128	144	144
TE	13.3 ms	13.3 ms	13.0 ms
Flip angle	30°	30°	30°
Receiver bandwidth	±5.68 kHz	±15.63 kHz	±62.5 kHz
TR	27.0 ms	26.2 ms	235 ms
Echo train length	n/a	n/a	16
Ramp sampling	n/a	n/a	Off
Receive coil	Body	Body	Body
Multi-phase delay time	0.05 s	2 s (default)	0.5 s (default)
No. of phase images	5	5	5

*n/a* not available

For each scenario, current densities were evaluated and the maximum gradient strengths *g*_max_ calculated from *B*_ref_ and *B*_ec_ using spherical harmonic decomposition [28] and were compared to the theoretical gradient strength of 49.5 mT/m. Therefore, the magnetic fields, or more precisely the *z* component of the magnetic flux density *B*_*z*_ was approximated by a truncated spherical harmonic series [33] inside an imaging volume of 20 cm diameter located at the iso-center of the *y*-gradient coil which is referred to spherical harmonic decomposition. The expansion into spherical harmonics contains spherical coordinates, Legendre polynomials and associated Legendre functions, and spherical harmonic coefficients. In the area of gradient coil design, the spherical harmonic coefficients represent the field strength in T/m^*n*^. Here, the magnetic fields were extracted and decomposed into the linear order term represented in Tesla per meter.

### Experimental setup

The ultrasound applicator of the InSightec ExAblate 4000 Neuro system (InSightec Ltd., Tirat Carmel, Israel) consists of a hemispherical 1024-element phased-array transducer operating at 650 kHz [4]. The transducer setup is interfaced to the MR scanner and is integrated into a patient table that can be docked to the MR system (Fig. 1c). Here, instead of the clinical transducer setup, a transducer mockup was positioned at the iso-center of the MR system, arranged in a manner similar to the real setup. The mockup consisted of a plastic hemisphere (Plexiglas® with 2-cm flange, Zeigis) 30 cm in diameter. In its center, a high-resolution quantification phantom (ADNI, Alzheimer’s Disease Neuroimaging Initiative phantom, Magphan, EMR051, Phantom Laboratory, Salem, NY, 2006) [36] was positioned. The superior end of the hemisphere and the ADNI phantom was sealed, and the inside of the hemisphere was filled with demineralized water to represent the water bolus of the clinical setup [4]. The alternative ground plane segmentation patterns previously characterized by FEM simulations were implemented using a thin copper foil (Scotch 1181, 3 M, MN, USA, 0.07 mm thick, 9 mm width) applied to the outside of the plastic hemisphere (Fig. 3).

For all experiments, the mockup phantom was unheated and a room temperature of 22 °C was assumed. For both sequences, the integrated body coil of the MR system was used to transmit the RF signal and receive the MR signal. The different setups were positioned as identically as possible at the iso-center of the MR scanner. Two-dimensional multi-phase SPGR and EPI images were acquired for geometric distortion analysis and temperature maps in the axial, sagittal, and coronal planes were computed. Table 1 shows the respective MR imaging protocol parameters, chosen after considering the trade-off between achieving good EPI image quality and adhering to the SPGR protocol typically used in the clinic. Some parameters were also adapted to simplify phase and frequency sampling.

### Data analysis

#### Geometric distortion quantification

The fine structures of the ADNI phantom enabled the use of regularized non-rigid registration based on a multi-resolution optical flow [37]. This algorithm generates vector maps of the geometrical distortions in all spatial directions in millimeters. In the experiments, the mockup transducer was positioned at the iso-center of the MR system and the SPGR and EPI images were acquired. To scan the next pattern, the mockup phantom was removed from the scanner, the ground plane was changed, and the phantom was positioned at marked positions at the iso-center of the MR scanner again. Despite careful calibration when installing the transducer mockup, changes in the phantom for the different copper patterns caused some minor spatial deviations. For this reason, the EPI images were registered to the significantly less distorted SPGR images of the same assembly, rather than to a standard reference image. The so-obtained geometrical distortion maps still differ due to the variable position of the excited slices; the variation caused by the copper shells should however be more significant.

Prior to registration, all magnitude images were masked to ensure that only (i) the area of the ADNI phantom and water bolus and (ii) regions with a high image intensity gradient are considered. Masks were generated for each scan axis individually and calculated by extracting the image intensity gradient of the magnitude image. A threshold number defines which points of the image intensity gradient were included into the masks. This threshold was selected such that the same amount of voxels could be guaranteed for all patterns. The resulting voxel numbers are listed in the figure captions. The means and standard deviations of the masked distortion maps were calculated to quantitatively compare the geometrical distortions of the different patterns.

#### Temperature map quantification

For each transducer ground plane setup and imaging plane, five consecutive phase images were acquired in the SPGR and EPI protocols to compute the corresponding temperature map with subsequent image acquisitions. For SPGR and EPI, the fourth phase image was defined as the baseline image *φ*_*T*0_ and subtracted from the fifth phase image *φ*_*T*_ according to Eq. (1) to calculate temperature maps with a temperature coefficient of *α* = −0.01 ppm/°C [38] (see discussion for details on why the fourth phase image has been chosen). As the phantom is unheated, the expected temperature values should be close to zero. The real transducer creates a hot spot in the sub-thalamus of the brain. Hence, certain regions of interest were selected for each copper pattern and imaging plane, respectively, such that (i) high SNR could be guaranteed and (ii) the central regions of the ADNI phantom were covered. The voxels inside the regions were used for the temperature map calculation. This means that for the sagittal and coronal case, voxels in the upper and lower part of the ADNI phantom were omitted, whereas the axial case remains almost unchanged. To avoid plastic structures within the selected regions of interest, voxels with a high image intensity gradient were excluded. Both image registration and temperature map calculations were performed using Matlab (Mathworks Inc., Natick, MA).

#### Geometrical distortion and temperature map evaluation

To provide an overview of the geometric distortion and temperature map characteristics for each copper pattern, $\frac{1}{\sigma }$ (*σ* is the standard deviation) representing the relative SNRs of each temperature map were plotted against the means and standard deviations of the geometrical distortions. Only the standard deviations of the temperature maps were considered because they represent the temperature variability depending on the copper patterns, whereas the means stayed near to 0 °C for most cases. This was done for both EPI and SPGR and separately for the sagittal, axial, and coronal axes. The geometric distortions are illustrated in the form of error bars, representing both the means and standard deviations. With this comparison, a mean near to 0 mm and a small standard deviation, but a high SNR, are favorable.

## Results

### Simulation

**Fig. 4:**
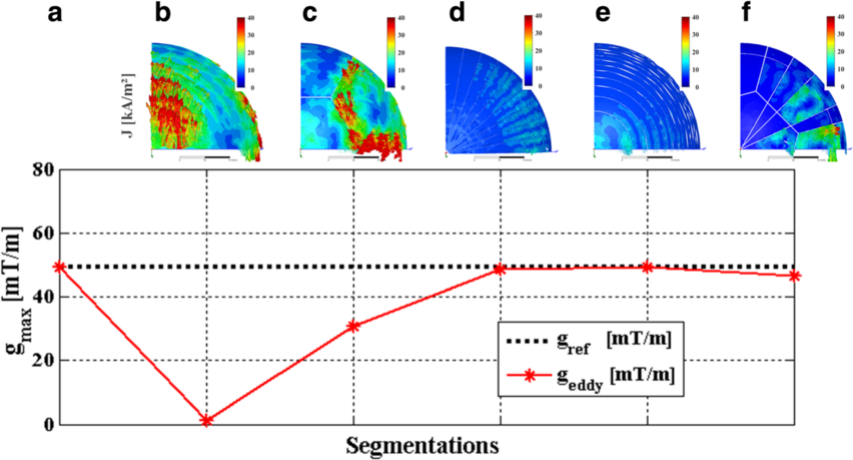
(*Top*) Densities of currents induced on the surfaces of the transducer ground planes when pulsing the *y*-gradient coil (scale 0 to 40 kA/m^2^). Current densities are plotted for patterns (**b**) to (**f**). Only one fourth of the model is shown given the symmetry boundary conditions in the FEM simulations. (*Bottom*) Calculated maximum gradient strengths on a 20-cm sphere inside the FOV of the gradient model, plotted for all segmentation patterns described in Fig. 3 (reference gradient strength *g*_ref_ = 49.44 mT/m)

The top row of Fig. 4 shows the current densities induced on the copper surface for each of the five simulated scenarios (b) to (f). The simulation images are scaled from 0 to 40 kA/m^2^ and only one fourth of the model is displayed owing to the symmetry boundary conditions used to accelerate FEM simulation times. The specified gradient strength of 49.5 mT/m as in the reference scenario was compared with the gradient strengths achieved by the individual copper patterns. Scenario (b) showed strong eddy currents towards the top of the hemisphere. The maximum magnetic field inside a 20-cm FOV became almost zero; hence, the un-segmented hemisphere acted like a shield (Fig. 4, bottom). For pattern (c), the currents due to segmentation occurred further to the outside of the copper hemisphere but remained strong because of the large segment areas. With segmentation into seven parts, the maximum gradient strength decreased by 37.6 % relative to (a) and strong non-linearity in the computed field map was observed. The star pattern in (d) resulted in a field reduction of 1.4 % relative to (a), whereas the reduction was 0.6 % with the ring pattern (e). With the ring and star pattern (f), the gradient field strength decreased by 5.6 % owing to an increased shielding effect.

### Experiment

The sagittal, axial, and coronal imaging axes exhibited different geometrical distortion and temperature characteristics and are therefore listed separately. For all axes, the geometric distortion map of the reference case (a) represents the difference in geometric distortion between SPGR and EPI, which is based on the pulse sequence and system characteristics but unrelated to geometric distortions due to eddy currents on the transducer ground plane. The means and standard deviations of the geometric distortion maps of the patterns (b) to (f) were compared with those of the reference scenario (a), assuming that further deviations in the difference between SPGR and EPI are mainly related to the additional copper patterns, less significant deviations between the individual patterns is caused by the variable position of the respective excited slice.

**Fig. 5:**
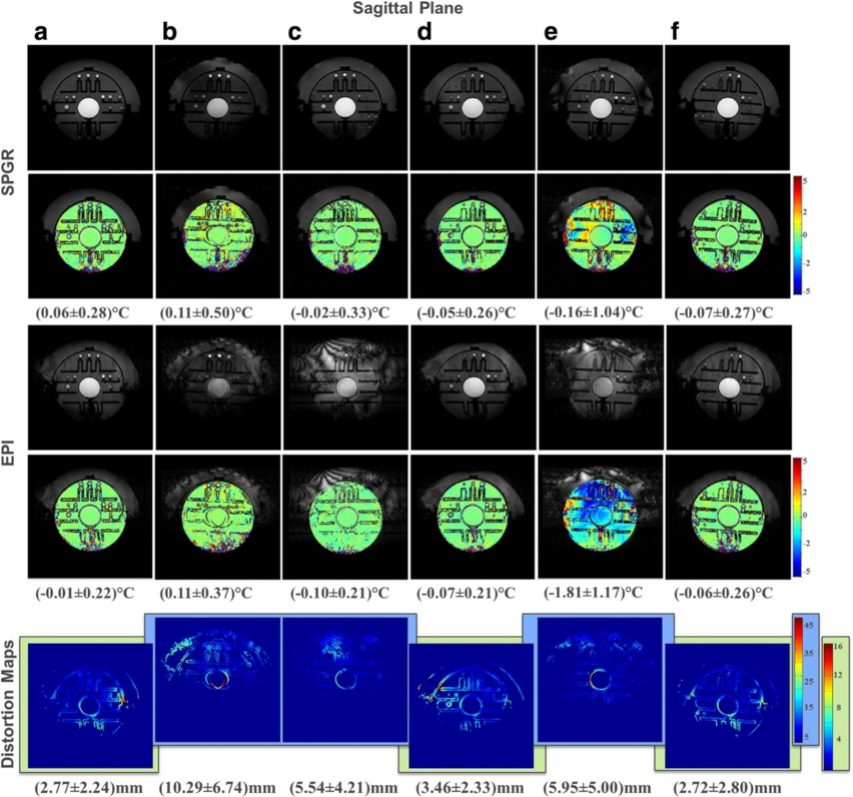
(*Top*) Sagittal images of patterns (**a**) to (**f**) in SPGR with calculated temperature maps. (*Middle*) Sagittal images of patterns (**a**) to (**f**) in EPI with calculated temperature maps. The mean and standard deviations were calculated on 2585 voxels of predefined region of interest. (*Bottom*) Geometric distortion maps created by masks to generate about 5000 voxels. Two different color scales are used to facilitate comparison

**Fig. 6:**
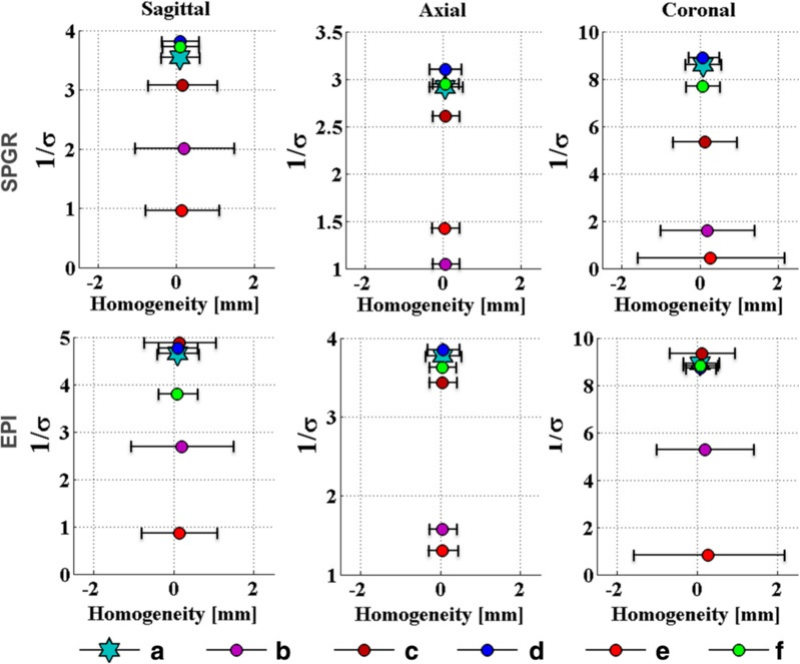
SNR expressed as 1/*σ* (ordinate) plotted against the standard deviation of geometric distortion expressed in millimeters (abscissa) in SPGR (*top*) and EPI (*bottom*) for the sagittal (*left*), axial (*center*), and coronal (*right*) planes for all scenarios (**a**) to (**f**). The desired design space comprises high SNR and small geometric distortion error bars centered at 0 °C

Figure 5 summarizes the results of the sagittal plane showing the geometric distortion maps (bottom row), the temperature maps inside the ADNI phantom calculated for SPGR (top row) and EPI (middle row) for all patterns. Within the geometric distortion maps, the setups (b), (c), and (e) showed much higher means and standard deviations than cases (a), (d), and (f) and were thus scaled differently for better visualization (blue background). The star pattern (d) (3.46 ± 2.33 mm) and star-ring setup (f) (2.72 ± 2.80 mm) showed smaller distortions than the seven-segment sheet (c) (5.54 ± 4.21 mm) or the ring pattern (e) (5.95 ± 5.00 mm) and were both comparable to the reference scenario (a) (2.77 ± 2.24 mm). However, the star-ring was assumed to achieve the best result because its distortions were consistently smaller, although the conclusion could be influenced by the mentioned variation in positioning of the phantom and hence excited slice. The top and middle rows of Fig. 5 show the SPGR and EPI temperature maps inside the ADNI phantom scaled to ±5 °C. The listed mean and standard deviations were computed in certain pre-selected regions as described in the ‘Temperature map quantification’ section; however, the complete temperature maps are shown in the graphs. The means and standard deviations stayed near 0 °C with small deviations for the ring pattern. The first column of Fig. 6 summarizes the relationship between the geometric distortion and SNR expressed by $\frac{1}{\sigma }$ for SPGR and EPI in the sagittal plane. These graphs show that scenarios (d) and (f) performed as well as the reference scenario (a) in terms of high SNR and small geometric distortions in SPGR, whereas in EPI, pattern (c) performed the best owing to its high SNR, albeit with higher standard deviations. For EPI, pattern (d) performed as well as the reference scenario (a).

**Fig. 7:**
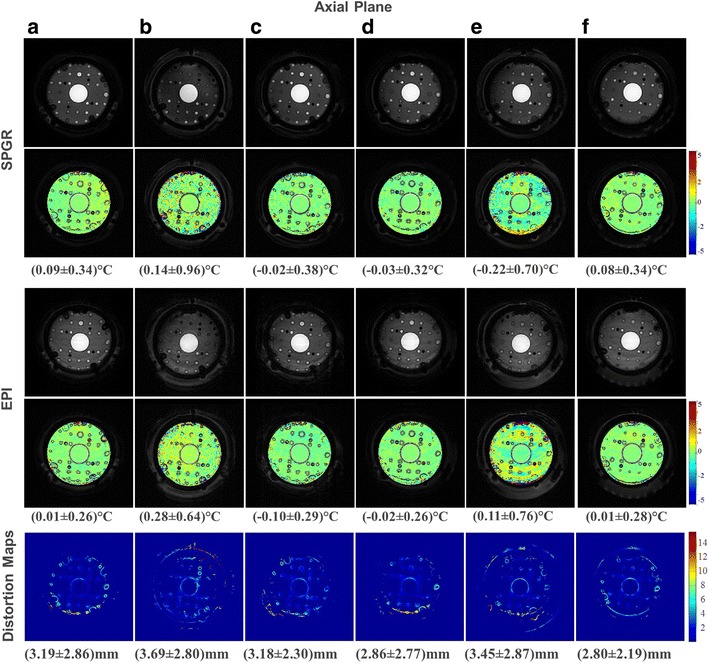
(*Top*) Axial images of patterns (**a**) to (**f**) in SPGR with calculated temperature maps. (*Middle*) Axial images of patterns (**a**) to (**f**) in EPI with calculated temperature maps. The mean and standard deviations were calculated on 7488 voxels of a predefined region of interest. (*Bottom*) Geometric distortion maps created by masks to generate about 2500 voxels

Figure 7 summarizes the results of the axial plane, showing the geometric distortion maps (bottom row) and the temperature maps for SPGR (top row) and EPI (middle row) for all patterns. All segmentation scenarios showed geometric distortions comparable to those of the reference scenario (a), suggesting that for an axially oriented slice selected towards the outer hemisphere, the transducer ground plane is not a critical imaging plane, although the conclusion could be influenced by the mentioned variation in positioning of the phantom and hence excited slice, which is also mirrored by the better performing cases (d) and (f) compared to the reference scenario (a). The second column of Fig. 6 shows the relationship between the SNR and geometric distortion for SPGR and EPI. For SPGR, scenarios (d) and (f) performed as well as the reference scenario (a). Scenarios (d) and (f) showed high SNR and small geometric distortions for EPI as well, although (c) also performed nearly as well as the reference scenario.

**Fig. 8:**
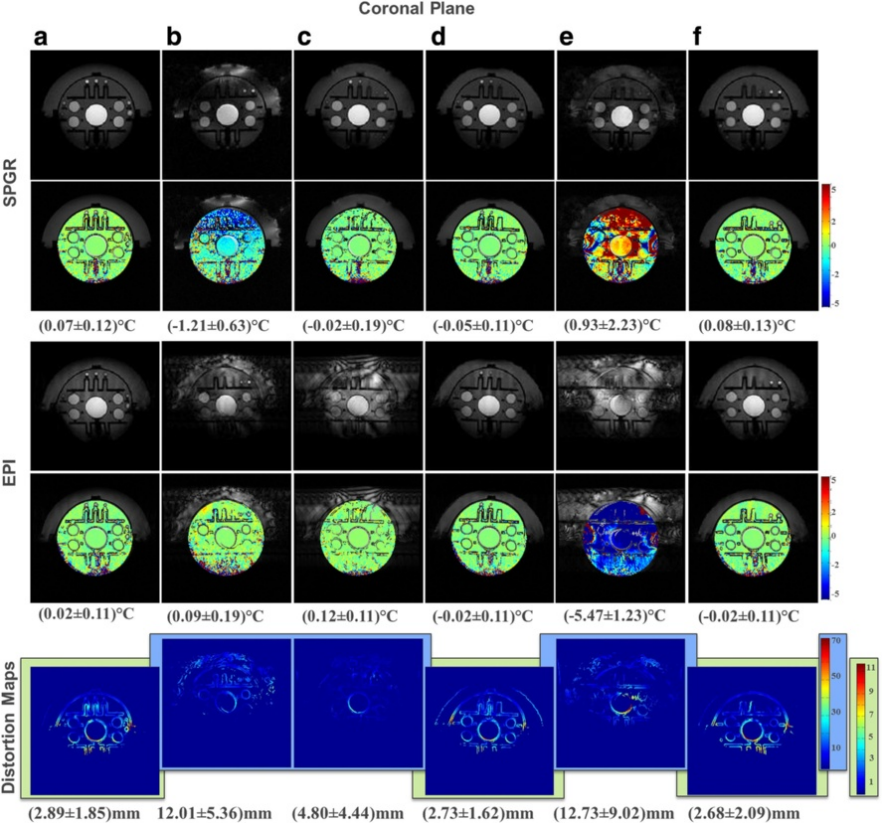
(*Top*) Coronal images of patterns (**a**) to (**f**) in SPGR with calculated temperature maps. (*Middle*) Coronal images of patterns (**a**) to (**f**) in EPI with calculated temperature maps. The mean and standard deviations were calculated on 1458 voxels of a predefined region of interest. (*Bottom*) Geometric distortion maps created by masks to generate about 4000 voxels. Two different color scales are used to facilitate comparisons

The coronal plane is summarized in Fig. 8, showing the geometric distortion maps (bottom row), the temperature maps for SPGR (top row) and EPI (middle row) for all setups. As in the sagittal case, the distortion maps of the copper patterns (b), (c), and (e) showed much higher means and standard deviations than those of the reference case (a), and hence were scaled separately. The star pattern (d) (2.73 ± 1.62 mm) and star-ring pattern (f) (2.68 ± 2.09 mm) performed markedly better than the seven-segment setup (c) (4.80 ± 4.44 mm) and comparably to the reference scenario (a) (2.89 ± 1.85 mm). Note that in the coronal plane, the star pattern has a slight advantage over the star-ring setup. The means of the SPGR and EPI temperature maps shown in the top and middle rows are again around 0 °C for patterns (a), (c), (d), and (f). However, those of the SPGR temperature map of the solid sheet (b) (−1.21 ± 0.63)°C and the ring setup (e) (SPGR (0.93 ± 2.23)°C, EPI (−5.47 ± 1.23)°C) show large deviations.

The relationship between the geometric distortion and SNR for the coronal case is summarized in the third column of Fig. 6 for SPGR and EPI. Scenarios (d) and (f) performed as well as the reference scenario (a) in SPGR, whereas in EPI, patterns (c), (d), and (f) performed the best, albeit with high standard deviations for (c). Table 2 summarizes the results of this section, listing the best-performing transducer segmentations in terms of geometric distortions and temperature map calculations.

**Table 2:** Best-performing patterns for the different pulse sequences and imaging planes tested. The exclamation mark after (c) emphasizes the SNR problem discussed in discussion

	Geometric distortion quantification	Geometric distortion vs. temperature mapping	Geometric distortion vs. temperature mapping
		SPGR	EPI
Sagittal	(f)	(d) (f)	(c) (!) (d)
Axial	(a)–(f)	(d) (f)	(d) (f) (c)
Coronal	(d)	(d) (f)	(c) (!) (d) (f)

## Discussion

The simulations in this study indicate that the ring pattern (e) delivered the best results with respect to shielding effects and the maximum gradient strength calculation. However, the experiments showed that the ring pattern performed worst with respect to geometric distortion and temperature map quantification in all imaging planes. One explanation is that the particular combination of gradient axes pulsed for the prescribed pulse sequence together with the orientation of the transducer patterns in the MR system has a strong impact on how the induced currents propagate on the surface of the rings. Only the *y*-gradient was simulated, whereas the EPI blips and readout gradient waveforms were played on the transverse *and* longitudinal gradient system in the experiment.

Due to computational limitations, the simulation were based on a set of assumptions such that only one gradient axis was included. To minimize the difference between simulation and experimental outcome, the simulation model should include additional geometrical details of the MR gradient system: for example the *x*- and *z*-gradient coils, in addition, the thickness of the copper ground plane should be reduced to 2 μm, of course both to a computational limitation. If these steps allow for a reproducible prediction between simulation and experiment, future work on the simulation phase could be to study the influences of the whole EPI frequency components. Here, the evaluation focused on the given clinical set of EPI sequence parameters slightly changed for an optimized image quality. It is suggested to additionally simulate a bandwidth of frequencies from a typical EPI frequency range of 0.1 to 5 kHz. With this, the eddy current duration parameter is described more generally. In tcMRgFUS, the transducer can only be changed by ±4 mm along the *z*-axis and ±1 cm in the *y*- and *x*-axes. To generally describe the associated eddy current amplitude changes, it is suggested to include the offset positions in the segmentation design. In addition, the *z*-gradient field change may also have induced currents on the ring structures, leading to the poor qualitative overlap between simulation and experimental results for this structure. We note that the copper patterns used in the experiment differed from those simulated as (i) the star and ring patterns had 4 and 1 segments less, respectively, and (ii) the star-ring pattern was established by further segmenting the star pattern and not the seven-segment sheet as in the simulation. The thickness of the copper material in the simulation differed from that in the experiment. The resistance of the copper was measured to be in the range of 0.2 Ω, although a concrete value could not be determined. Furthermore, the solid copper pattern was fixed on the inside of the plastic shell and therefore remained in direct contact with the demineralized water. This might influence the propagation of the induced eddy currents in a different way than in the other setups. To summarize, the FEM simulations indicated that the imaging artifacts resulted from eddy currents induced in the copper ground plane. The simulations helped to achieve a visible representation of the induced currents on the surface of the copper ground plane, and the simulated gradient field strengths helped to identify segmentation patterns that minimized the eddy current flow with respect to the actively pulsed gradient coil. Consequently, by considering the discussed items, we expect a more user specific set of EPI parameters to be detectable.

Geometric distortion quantification was achieved by registering the EPI images to the SPGR images to obtain a relative error expressed in millimeters. In this scenario, SPGR is seen as the ground truth as SPGR is used in clinical treatment. The geometric distortion maps of the different setups can be compared directly with each other because imaging parameters such as the SNR have a negligible influence on the image contrast masks of the registration algorithm.

The overall performance of the star setup indicates that this segmentation structure is superior also with respect to the clinical pattern (c). Nevertheless, it was found that (e) and even the higher segmented pattern (f) showed higher geometric distortions than (d) in the axial and sagittal plane. This illustrates that certain pattern orientations in relation to the gradient system can actually increase geometric distortions even with a higher degree of segmentation. Therefore, a star pattern with a degree of segmentation as high as possible and an appropriate orientation is supposed to achieve the best possible results in concerns of geometrical distortions.

Temperature maps were extracted according to [1] by using the fourth phase image as the baseline image. The first of the five phase images showed higher SNRs than the following four phase images. This suggests that the system is stable and quickly settled into a steady state. Given that the change in SNR between the fourth and fifth images is less than 1 %, we regard the images used to calculate the temperature map as appropriately chosen.

The performance of the segmentation pattern was judged by setting the geometric distortion analysis into relation with the SNR evaluation (Fig. 6), expressed by 1/*σ*. The results were compared to the reference pattern (a). Here, upper and lower specification limits were not defined, as would be required within a design process. Additionally, for each scenario, the mockup model was positioned slightly different. Hence, the mean and standard deviations of the temperature maps and hence the decision on which scenario performed best might be influenced by the mentioned variation in positioning of the phantom and hence excited slice.

We also note that the sagittal and coronal planes show noisy areas towards the superior end. This could be explained by assuming that the shimming was optimized based on a MR signal collected from areas that contained the water bolus; hence, areas with no water bolus might show lower SNRs. Consequently, these areas were excluded from the temperature map calculation.

Here, only eddy current effects induced in the transducer ground plane were considered. Other sources of image distortion that arise from the motion of water pumped through the water bolus for scalp cooling or motion due to vibration have not been considered. In addition to optimizing the hardware components of the transducer, future studies could concentrate on compensating for spatially varying eddy currents in image reconstruction by calibrating the gradient field with static magnetic field measurements in real time. This can be achieved, for example, with the help of magnetic field monitoring [39], by a pre-calibration technique described by Duyn et al. [40], or by applying higher-order eddy current field correction algorithms as described by Xu et al. [30]. To further increase the temporal resolution, parallel imaging could be considered.

A further open question targets the fact whether a segmentation of the transducer ground plane additionally increases the RF-related heating properties, which is not addressed within this work. The used specific absorption rate value for the SPGR and EPI sequences were 0.53 W/kg (all three planes) and 0.02 W/kg (sagittal, coronal) and 0.08 W/kg (axial), respectively.

## Conclusions

In conclusion, gradient-induced eddy currents in the tcMRgFUS transducer ground plane can dramatically degrade EPI image quality. The most critical impact of our results relates to the method used to quickly and efficiently down-select transducer designs for experimental prototyping. The root cause of EPI image distortion was investigated with FEM simulations and verified by experiments on phantoms. In simulations, artifact-causing eddy currents were reduced by increasing the segmentation of the transducer ground planes. The experiments showed that the degree of transducer ground plane segmentation, the pattern of segmentation with respect to the orientation of the pulsed gradient coil, and pulse sequence characteristic determine image quality and accuracy of the temperature map. In this particular test environment, the star pattern showed the best overall performance in terms of mitigating eddy current-induced geometric distortions due to fast-switching gradients, and producing accurate MR thermometry maps.
